# Topological feature-driven TabPFN model for prediction of enlarged hemorrhage and edema after tumor resection in meningiomas

**DOI:** 10.3389/fmed.2026.1808831

**Published:** 2026-05-06

**Authors:** Wenjing Han, Guirong Tan, Lijia Li, Zhenyang Feng, Chen Zhou, Xiang Liu, Lingjing Hu

**Affiliations:** 1 Key Laboratory of Media Audio and Video of Ministry of Education, Communication University of China, Beijing, China; 2 School of Medical Technology, Capital Medical University, Beijing, China; 3 Advanced Neuroimaging Laboratory, Yuebei People’s Hospital Affiliated to Shantou University Medical College, Shaoguan, Guangdong, China; 4 Department of Radiology, Yuebei People’s Hospital Affiliated to Shantou University Medical College, Shaoguan, Guangdong, China

**Keywords:** enlarged hemorrhage and edema after tumor resection, meningiomas, peritumoral edema, SHAP, topological data analysis

## Abstract

**Introduction:**

Enlarged hemorrhage and edema after tumor resection (EHETR) is a serious postoperative complication in meningiomas. The peritumoral edema (PE) region was reported to be associated with EHETR. Topological data analysis (TDA) has recently emerged as a novel approach providing a multiscale characterization of structural organization. This study aims to assess the feasibility of using topological features extracted from the PE region to predict EHETR.

**Methods:**

We retrospectively enrolled 161 patients with meningiomas, of whom 79 (49.1%) developed EHETR. Multiscale topological features were extracted from the PE regions on preoperative MRI sequences, including contrast-enhanced T1-weighted imaging (T1CE), T2-weighted imaging (T2WI), and apparent diffusion coefficient (ADC) maps, using cubical persistent homology. Feature selection was performed using the least absolute shrinkage and selection operator (LASSO) within a nested five-fold cross-validation framework (5-fold outer loop and 3-fold inner loop). Subsequently, predictive models were constructed using the Tabular Prior-data Fitted Network (TabPFN). Model performance was evaluated using receiver operating characteristic (ROC) analysis, calibration curves, and decision curve analysis. Model interpretability was further assessed using SHapley Additive exPlanations (SHAP) to quantify feature contributions.

**Results:**

The ADC-based topological model exhibited superior discriminative performance, achieving a mean area under the receiver operating characteristic curve (AUC) of 0.80 (95% CI: 0.71–0.89) in the validation set, compared with models based on T1CE (AUC: 0.74) and T2WI (AUC: 0.70). DeLong tests further confirmed that the ADC-based model significantly outperformed models based on T1CE and T2WI (DeLong, *P* < 0.001). SHAP analysis highlighted persistence landscape features, particularly those from the H_1_ (loops) and H_2_ (cavities) homology dimensions as the primary drivers of prediction, suggesting that higher-order topological features from the PE region may be key contributors to EHETR.

**Conclusion:**

The topological features derived from the PE region can predict EHETR in patients with meningiomas as a novel computational imaging framework.

## 1 Introduction

Meningiomas are the most common primary intracranial tumors in adults, and surgical resection remains the primary treatment (1). Intracranial hemorrhage and cerebral edema are major postoperative complications. Previous studies showed that the incidence of postoperative hemorrhage in meningiomas was 33.33% (2). A recent study found postoperative cerebral edema exacerbation in 44.12% of patients (3), and another investigation reported the incidence of progressive edema and hemorrhage as 49% (4). Enlarged hemorrhage and edema after tumor resection (EHETR) is a serious postoperative complication in meningiomas and can lead to severe neurological deficits, prolonged hospitalization, and even mortality (5, 6), indicating the clinical importance of accurate preoperative prediction of EHETR. The occurrence of EHETR was reported to be associated with multiple risk factors, including peritumoral edema (PE) (7, 8). Previous studies showed that postoperative hemorrhage is significantly increased in patients with PE, and that PE can cause clinical symptoms and complicate surgery, both of which are closely related to poor postoperative prognosis (9, 10). Therefore, the PE area is a new research field for exploring EHETR. Thus, this study focuses on developing a predictive model based on PE features to predict EHETR. Literature specifically addressing EHETR after meningioma resection remains limited, and existing related studies have mainly relied on clinical variables, conventional imaging findings, or radiomics-based approaches (3, 4). Traditional clinical models are limited by a relatively small number of features and rely on clinicians’ subjective judgment, making it difficult to achieve stable and quantitative assessment (11). Although radiomics models can extract high-throughput features, they are typically based on the entire volume of interest and therefore are limited in their ability to reflect intratumoral heterogeneity (12).

In contrast, topological data analysis (TDA) provides a novel mathematical framework for characterizing spatial organization and multiscale structural heterogeneity within the region of interest (13–16). As a core tool of TDA, persistent homology constructs topological representations across varying filtration thresholds and tracks the emergence and disappearance of connected components, loops, and three-dimensional voids, enabling multiscale characterization of lesion structure (17, 18). This approach yields compact and structurally meaningful features that are robust to noise and summarize lesion architecture in a stable manner across scales, making it well suited for studies with limited sample sizes and high dimensional data.

Notably, TDA has demonstrated utility in brain imaging studies, including brain network connectivity analysis (19, 20), age prediction in datasets related to Alzheimer’s disease (21), and glioma studies such as tumor grading and molecular biomarker prediction (22). However, topological studies in meningiomas remain limited. Friconnet et al. reported that fractal dimension and topological skeleton features extracted from preoperative contrast-enhanced MRI were associated with histologically aggressive meningiomas (23), but the analysis focused on boundary shape rather than characterizing the three-dimensional tumor structure using persistent homology. In addition, existing topological studies in meningioma have not involved the PE region, which is clinically relevant to postoperative hemorrhagic and edematous complications. We hypothesize that topological features derived from the PE region can effectively predict EHETR in meningioma.

To our knowledge, no prior study has specifically investigated the PE region topological analysis in meningioma, nor has its association with postoperative hemorrhagic or edematous complications been explored. To fill these gaps, the present study investigates the feasibility of using topological features extracted from the PE region to predict postoperative EHETR in patients with meningiomas. A transformer-based Tabular Prior-data Fitted Network (TabPFN) model (24) is employed to support robust prediction in small sample, high dimensional settings, and SHapley Additive exPlanations (SHAP) analysis is used to improve interpretability and quantify the contributions of topological features.

## 2 Materials and methods

### 2.1 Patient selection and MRI acquisition

This study was approved by the Ethics Committee of Yuebei People’s Hospital, and the requirement for informed consent was waived owing to its retrospective design. We retrospectively reviewed 227 patients with pathologically confirmed meningiomas who underwent surgical resection at our institution between January 2018 and September 2023. Patients were included according to the following criteria: (1) availability of preoperative brain MRI; (2) no history of radiotherapy, chemotherapy, or other treatments prior to MRI; (3) continuous postoperative follow-up with CT and/or MRI within 1 month after surgical resection; (4) complete clinical data. Patients were excluded if they met any of the following conditions: (1) presence of coagulation dysfunction; (2) coexistence of other major diseases that could significantly influence brain imaging findings, such as severe cerebrovascular disorders; (3) inadequate image quality, including pronounced artifacts or incomplete MRI acquisition, or imaging data from which reliable topological features could not be extracted.

Preoperative brain MRI examinations were conducted according to standardized clinical scanning protocols. The acquired sequences comprised contrast-enhanced T1-weighted imaging (T1CE), axial T2-weighted imaging (T2WI), and diffusion-weighted imaging (*b* = 0 and 1,000 s/mm^2^), from which apparent diffusion coefficient (ADC) maps were automatically generated.

### 2.2 The definition standard for EHETR

EHETR was defined when any of the following criteria were met: (1) Newly developed sheet-like or finger-like cerebral edema detected after surgery, with a maximum diameter ≥ 2 cm. (2) In patients without preoperative PE, newly developed flaky, finger-like, or annular cerebral edema observed after surgery, with a maximum diameter ≥ 2 cm at the tumor cavity or surgical region on the same imaging slice compared with preoperative or postoperative day 1 imaging. (3) In patients with preoperative PE, postoperative lamellar, finger-like, or annular cerebral edema showing an increase in maximum diameter ≥ 2 cm on the same imaging plane compared with preoperative or postoperative day 1 imaging. (4) Progressive cerebral edema demonstrated by serial postoperative CT and/or MRI examinations. (5) Postoperative intracranial hemorrhage within the surgical field with a hematoma volume **>** 50 ml. (6) Progressive increase in cerebral hemorrhage detected on serial postoperative CT and/or MRI scans. (7) Requirement for reoperation for hematoma evacuation and/or decompressive craniectomy. (8) Clinical deterioration attributable to hemorrhage or edema, including decreased level of consciousness, anisocoria, or death.

### 2.3 ROI segmentation

**FIGURE 1:**
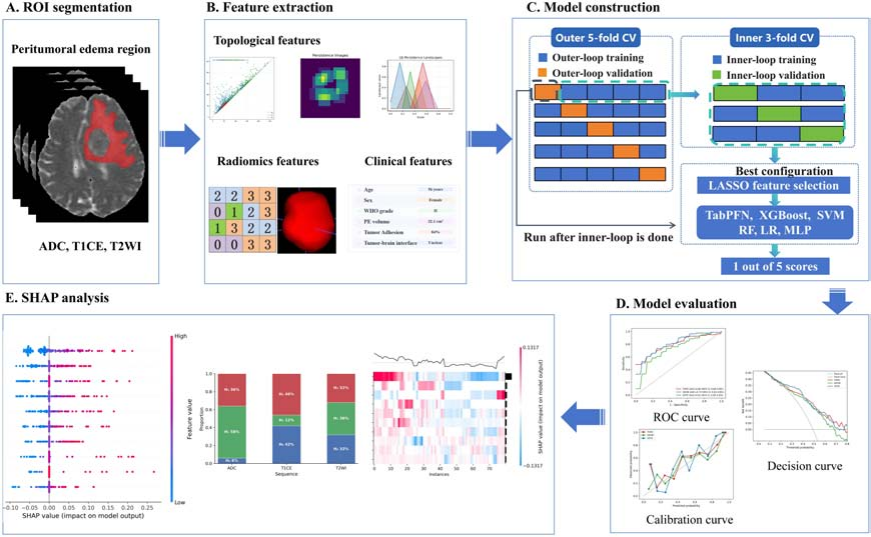
Workflow of the proposed framework for predicting postoperative EHETR using topological features. **(A)** ROI segmentation. **(B)** Topological feature extraction. **(C)** Model construction. **(D)** Model evaluation. **(E)** SHAP analysis.

For each patient, regions of interest (ROIs) corresponding to the PE region were manually delineated using ITK-SNAP (version 3.8.0). ROI delineation was independently performed by two radiologists in a double-blind manner, with both readers blinded to the clinical and outcome information. Inter-observer agreement was assessed using the Dice similarity coefficient (DSC), which was calculated separately for the ADC, T1CE, and T2WI sequences. The mean ± standard deviation DSC values were 0.92 ± 0.06, 0.91 ± 0.01, and 0.89 ± 0.01, respectively. In cases of discrepant segmentation, the final ROI was determined by the more experienced radiologist. The overall methodological workflow of this study is illustrated in Figure 1 and includes ROI segmentation, feature extraction, model construction, model evaluation, and SHAP analysis.

### 2.4 Clinical characteristics

Clinical and perioperative characteristics were retrospectively collected from the electronic medical records, operative reports, and imaging data. The collected data included age, sex, World Health Organization (WHO) grade, tumor location, systolic blood pressure, diastolic blood pressure, glucose, tumor volume, edema index, tumor consistency, intraoperative blood loss, operative time, and extent of resection.

Meningioma histopathological grading was assessed according to the 2021 WHO Classification of Tumors of the Central Nervous System. Tumor location was recorded as supratentorial, infratentorial, or sellar region. Tumor consistency was categorized as soft, moderate, or hard. The edema index was included as an imaging indicator to evaluate the extent of peritumoral edema and was calculated as (Vtumor + Vedema) / Vtumor (25). Extent of resection was recorded according to Simpson grade and dichotomized as total resection or subtotal resection for analysis (26).

### 2.5 Topological feature extraction

To compute persistent homology at the voxel level in medical images, we adopted cubical persistence, a formulation specifically designed for data defined on regular grids. In cubical persistence, a three-dimensional medical image is represented as a cubical complex, where each voxel is treated as a three-dimensional cube together with its associated faces, edges, and vertices. This representation allows topological structures to be defined directly on the native image lattice, without the need to construct point clouds or surface meshes. As MRI data are inherently represented on a regular voxel grid, cubical complexes provide a natural and computationally efficient framework for persistent homology analysis of volumetric MRI data. Cubical persistence homology was implemented using the giotto-tda library (version 0.6.0; https://github.com/giotto-ai/giottotda). All images were resampled to an isotropic voxel size of 1 mm. Linear interpolation was used for image resampling, whereas nearest-neighbor interpolation was used for ROI masks. Voxel intensities within the ROI were then normalized to the range 0-255 to improve comparability across patients. The workflow consisted of three main steps for topological feature extraction.

(1) Filtration construction. Filtration is a core concept in TDA and provides the foundation for computing persistent homology by constructing a sequence of nested topological spaces across multiple scales. In this study, a sublevel filtration was applied to cubical complexes, in which voxels with intensity values less than or equal to a threshold t were progressively activated. In practice, sublevel filtration was constructed based on voxel intensities normalized within the ROI. Accordingly, the filtration threshold t ranged from 0 to 255, and voxels with filtration values less than or equal to t were progressively included in the cubical complex as t increased.

For three-dimensional medical images, this procedure defines a multiscale family of cubical complexes in which topological structures emerge and disappear as the threshold varies. Specifically, topological structures across three homology dimensions are characterized in three-dimensional images. H_0_ represents connected components, reflecting the fragmentation versus confluence of activated regions as components appear and merge with increasing thresholds. H_1_ corresponds to loops or tunnels, capturing ring-like hole configurations formed by surrounding voxels and their evolution across thresholds. H_2_ captures enclosed cavities, describing fully enclosed three-dimensional voids. Together, these dimensions provide complementary characterizations of spatial organization in volumetric data, from basic connectivity to higher-order structural complexity.

**FIGURE 2:**
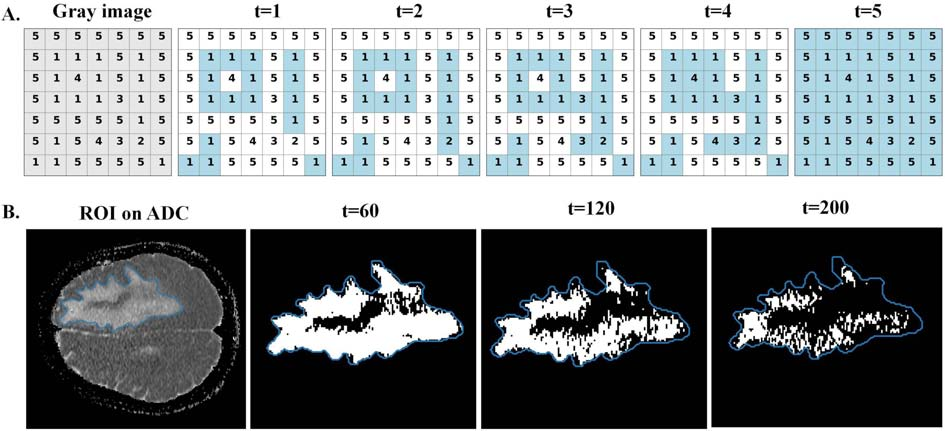
Sublevel-set filtration examples. **(A)** Sublevel-set filtration of a grayscale matrix at thresholds *t* = 1–5, illustrating the progressive activation of pixels and the resulting changes in topological structures. **(B)** Sublevel-set filtration of an MRI region of interest (ROI) on ADC, shown at thresholds *t* = 60, 120, 200.

As shown in Figure 2A, a two-dimensional gray-level matrix with gray-level values ranging from 1 to 5 is used as a schematic illustration of the sublevel filtration. In the illustration, activated pixels are highlighted in blue, whereas inactive pixels are shown in gray. At *t* = 1, only pixels with gray level 1 are activated, forming four spatially disconnected foreground regions. Each region constitutes a connected component, resulting in four H_0_ structures. Meanwhile, the central ring-shaped activated pixels form a closed loop that encloses an inactive region, yielding a single hole and thus one H_1_ structure. At *t* = 2, pixels with gray level 2 become activated; however, the newly activated pixels do not change the connectivity among existing foreground regions, nor do they create new holes or fill the existing holes. Therefore, the H_0_ and H_1_ configurations remain unchanged, and no new birth or death events occur at this threshold. At *t* = 3, some of these newly activated pixels create connections between previously disconnected foreground regions, leading to the merging of two connected components. This merging event corresponds to the death of one H_0_ structure. Meanwhile, the central ring-shaped activated pixels continue to enclose an inactive region, and the hole remains unchanged, so the number of H_1_ structures is preserved. When the threshold reaches *t* = 4, the pixel within the enclosed region becomes activated, filling the hole and causing the death of the H_1_ structure. Finally, at *t* = 5, the remaining pixels are activated, causing all regions to merge into a single connected component and producing additional H_0_ death events, while the last surviving component persists to the end of the filtration. This example illustrates how persistent homology summarizes multiscale topological evolution by tracking birth and death events at specific thresholds.

Figure 2B shows a representative two-dimensional ADC slice for visualization. With intensity values normalized to the range of 0–255, increasing thresholds of *t* = 60, 120, 200 are applied, and pixels with intensities less than or equal to the threshold are activated. As the threshold increases, activated pixels progressively expand and merge within the ROI. Persistent homology was computed on the full three-dimensional ROI; tracking the birth and death of topological features across thresholds yields the persistence information.

**FIGURE 3:**
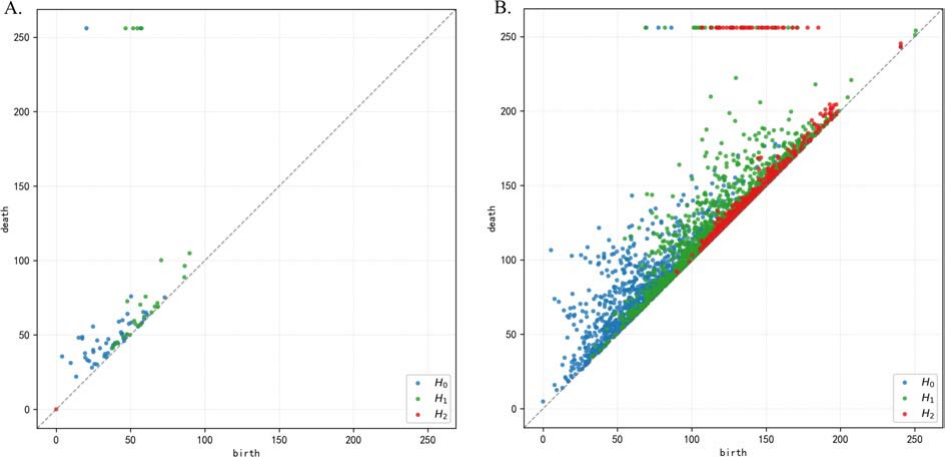
Persistence diagrams from representative regions with different levels of topological complexity. **(A)** Sparse distribution of persistence points. **(B)** Dense distribution of persistence points.

(2) Persistent homology computation. The filtration records the evolution of topological structures from birth to death, which is summarized in a persistence diagram representing the multiscale topological evolution, as shown in Figure 3. In a persistence diagram, each point represents a single topological structure in a given homology dimension, characterized by the threshold at which it appears (birth) and the threshold at which it disappears (death). The vertical distance of a point from the diagonal reflects its lifespan, defined as the difference between its death and birth values. Points close to the diagonal correspond to short-lived structures, whereas points farther from the diagonal indicate more persistent and stable structures.

Figures 3A,B show persistence diagrams from two representative regions with different levels of topological complexity. Overall, the number and distribution of points reflect the richness of topological events across the filtration. Figure 3A is relatively sparse, indicating fewer topological structures, with particularly few H_2_ points, suggesting limited cavity patterns and a simpler three-dimensional organization. In contrast, Figure 3B exhibits a markedly denser distribution of points across H_0_, H_1_, and H_2_, reflecting substantially richer multiscale topological activity.

(3) Vectorization of persistence diagrams. Each ROI produces a persistence diagram. Because the diagram is an unordered set of birth and death pairs and the number of points varies across samples, it cannot be used directly as input for standard machine learning models. Therefore, persistence diagrams need to be converted into fixed-dimensional feature representations for downstream analysis. This conversion is designed to preserve key multiscale topological patterns, including the strength and stability of structures across thresholds. To achieve this, we summarize the diagrams using complementary descriptors based on point distributions, function representations, and geometric organization on the birth and death plane, yielding multiple types of fixed-length topological features.

In this study, we employed seven families of topological features. For each feature family, features were extracted separately from the H_0_, H_1_, and H_2_ homology dimensions, corresponding to connected components, loop-like structures, and cavity-like structures, respectively. Specifically, these included: (i) Stats: counts of birth and death pairs and persistence summaries, including the number of persistence points, the sum, maximum, and mean of persistence lifetimes, as well as summary statistics of birth, death, and lifetime values; (ii) Entropy: dispersion of the persistence distribution; (iii) Betti curves: the number of active features as a function of threshold; (iv) Persistence landscapes: functional summaries of feature strength across scales, with 3 layers and 50 sampling bins; (v) Amplitude metrics: distance measures of persistence, including bottleneck-, Wasserstein-, and landscape-based measurements; (vi) Persistence images: gridded density representations on the birth-death plane, using a Gaussian smoothing width of 1.0 and a 20 × 20 grid, and further summarized by global statistics and 4 × 4 block-wise pooled features; and (vii) Heat-kernel signatures: diffusion driven multi-scale summaries, using a Gaussian smoothing width of 1.0 and a 20 × 20 grid, and likewise summarized by global statistics and 4 × 4 block-wise pooled features. These feature families capture complementary aspects of persistent homology, ranging from global summary statistics to functional and distribution representations. Aggregating features across all feature families and homology dimensions yielded a total of 717 quantitative topological features for each region of interest.

### 2.6 Radiomics feature extraction

For each ROI, 960 radiomics features were extracted using the PyRadiomics (https://github.com/AIM-Harvard/pyradiomics) library. All preprocessing and feature extraction settings were specified in a YAML configuration file to ensure reproducibility. The extracted features included: (1) first-order statistics, (2) shape descriptors, (3) texture features derived from gray-level co-occurrence, run-length, size-zone, and dependence matrices, and (4) features generated from wavelet-transformed images.

### 2.7 Model construction

Model development was conducted within a nested stratified cross-validation (CV) framework, with five-fold CV in the outer loop for performance evaluation and three-fold CV in the inner loop for feature selection and hyperparameter tuning, where stratification was based on EHETR occurrence to preserve class balance across folds. All preprocessing steps were performed using training data only within each split to avoid information leakage. Preprocessing of both radiomics and topological features included removal of features with more than 30% missing values, removal of low-variance ( < 0.001) and constant features, median imputation, and *z*-score normalization. Within each inner-loop training split, the least absolute shrinkage and selection operator (LASSO) was applied for feature selection. Three models were constructed based on the input feature sets: a TDA model using topological features, a radiomics (RAD) model using radiomics features, and a TDA-RAD model using the direct concatenation of topological and radiomics features.

For each feature strategy, all six candidate classifiers were refit on the corresponding outer-loop training set and evaluated on the outer-loop validation fold. (1) TabPFN is a pretrained Transformer model for tabular data that enables direct transfer to downstream tasks without conventional task-specific training. In this study, version 6.3.2 was used with default inference settings, without task-specific fine-tuning or additional hyperparameter optimization; therefore, conventional training hyperparameters such as learning rate and batch size were not applicable. (2) Multilayer perceptron (MLP) was evaluated using one- or two-hidden-layer architectures, with hidden-layer sizes ranging from 64 to 256 neurons. The L2 regularization coefficient ranged from 10^–4^ to 10^–3^, and the initial learning rate ranged from 5 × 10^–4^ to 10^–3^. Early stopping was applied. (3) Support vector machine (SVM) was evaluated with the penalty parameter C ranging from 0.1 to 5.0 and γ set to either “scale” or fixed low values. Probabilistic outputs were obtained using sigmoid calibration with three-fold cross-validation. (4) L1-regularized logistic regression (LR) was implemented using the saga solver. The inverse regularization strength C was explored over a range of 0.01–10.0. (5) Extreme Gradient Boosting (XGBoost) was evaluated using a lightweight grid with 100–500 trees, a maximum tree depth of 2 to 4, and learning rates from 0.03 to 0.1. Subsampling and feature subsampling ranged from 0.7 to 0.8, with additional regularization to control model complexity. (6) Random forest (RF) was included as a tree-based ensemble baseline. The number of trees ranged from 200 to 500, while candidate settings varied in tree depth, minimum leaf size, and feature subsampling.

### 2.8 Model evaluation

The primary evaluation metrics included the area under the receiver operating characteristic curve (AUC) and its 95% confidence interval (CI), as well as accuracy, sensitivity, and specificity. Model discrimination and calibration were assessed using receiver operating characteristic (ROC) and calibration curves, and clinical utility was evaluated using decision curve analysis. These metrics were calculated separately on the outer-loop training set and the corresponding outer-loop validation fold in each iteration of the outer 5-fold cross-validation and summarized as mean values across the five outer folds.

### 2.9 Statistical analysis

Clinical and demographic characteristics were compared between the EHETR and non-EHETR groups. Continuous variables were assessed for normality with the Shapiro-Wilk test, expressed as mean (standard deviation, SD) or median (interquartile range, IQR), and compared using the independent-samples *t*-test or Mann-Whitney U test. Categorical variables were summarized as counts with percentages and compared using the chi-square test or Fisher’s exact test. Differences in discriminative performance between models constructed from topological features derived from different MRI sequences were evaluated using DeLong’s test.

### 2.10 SHAP analysis

To interpret model predictions, SHAP was used to quantify the contribution of individual features. Among the evaluated classifiers, the best-performing classifier identified under the nested cross-validation framework was selected for SHAP analysis. SHAP values were computed for samples in each outer-loop validation fold using the corresponding fold-specific preprocessing pipeline and the trained classifier. A SHAP permutation explainer was applied to estimate feature contributions to the predicted probability of the positive class. SHAP values were aggregated across folds to obtain global feature importance, and the results were visualized using beeswarm and heatmap plots.

## 3 Results

### 3.1 Clinical characteristics

As shown in Table 1, compared with the non-EHETR group, patients in the EHETR group had significantly larger tumor volume, higher edema index, and greater intraoperative blood loss (all *P* < 0.05). No significant differences were observed in sex, age, WHO grade, tumor location, blood pressure, glucose, tumor consistency, operative time, or extent of resection between the two groups.

**TABLE 1:** Clinical characteristics of patients with EHETR and non-EHETR.

Characteristics	Whole cohort 161 (100%)	Non-EHETR 82 (51%)	EHETR 79 (49%)	*P*-Value
**Sex**				0.618
Female	117 (72.67)	61 (74.39)	56 (70.89)	
Male	44 (27.33)	21 (25.61)	23 (29.11)	
**Age (mean(SD))**	54.59 (11.02)	53.62 (11.24)	55.59 (10.76)	0.257
**WHO grade**				0.627
grade I/II (excluding atypical)	147 (91.30)	74 (90.24)	73 (92.41)	
Atypical grade II/grade III	14 (8.70)	8 (9.76)	6 (7.59)	
**Location**				0.084
Supratentorial	124 (77.02)	58 (70.73)	66 (83.54)	
Infratentorial	19 (11.80)	14 (17.07)	5 (6.33)	
Sellar region	18 (11.18)	10 (12.20)	8 (10.13)	
**Systolic blood pressure**	130 [120, 142]	131 [122, 140]	130 [120, 150]	0.981
**Diastolic blood pressure**	81 [76, 90]	82 [76, 90]	80 [78, 90]	0.889
**Glucose**	5.15 [4.63, 6.00]	5.09 [4.58, 5.85]	5.17 [4.71, 6.14]	0.158
**Tumor volume**	25,326 [11,211, 50,021]	18,280 [7,887, 38,491]	33,002 [18,503, 77,740]	< 0.001
**Edema index**	1.33 [1.00, 2.11]	1.00 [1.00, 1.60]	1.51 [1.11, 2.51]	< 0.001
**Tumor consistency**				0.151
Soft	28 (17.39)	18 (21.95)	10 (12.66)	
Moderate	89 (55.28)	46 (56.10)	43 (54.43)	
Hard	44 (27.33)	18 (21.95)	26 (32.91)	
**Intraoperative blood loss**	300 [150, 600]	300 [100, 500]	400 [200, 600]	0.014
**Operative time**	410 [290, 525]	405 [285, 525]	425 [290, 530]	0.480
**Extent of resection**				0.233
Total resection	147 (91.30)	77 (93.90)	70 (88.61)	
Subtotal resection	14 (8.70)	5 (6.10)	9 (11.39)	

Data expressed as n (%), mean(SD), or median [interquartile range, IQR], unless otherwise stated

### 3.2 Feature stability analysis

**FIGURE 4:**
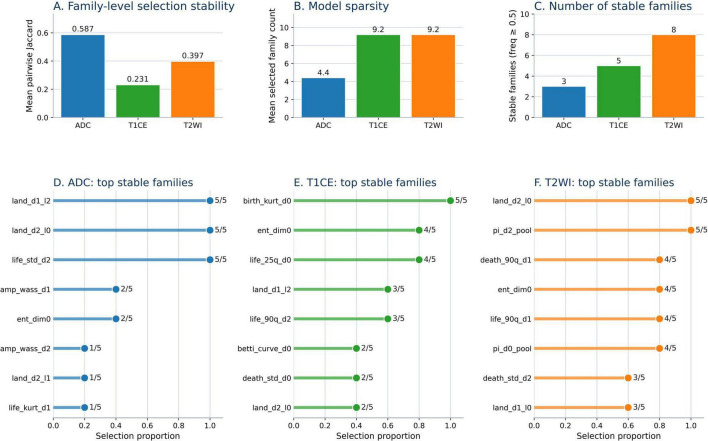
Comparative family-level feature stability across ADC, T1CE, and T2WI. **(A)** Mean Jaccard index of selected families. **(B)** Mean number of selected families. **(C)** Number of stable families. **(D–F)** Top stable families for ADC, T1CE, and T2WI, respectively.

Family-level stability was assessed using the selection frequency of each feature family across the 5 outer CV folds, together with the mean pairwise Jaccard similarity of selected family sets. The family-level analysis showed that ADC exhibited the highest stability among the three sequences (Figure 4). Specifically, ADC achieved the largest mean pairwise Jaccard similarity (0.587), compared with 0.397 for T2WI and 0.231 for T1CE (Figure 4A). Although ADC involved fewer selected families on average (4.4 vs. 9.2 for both T2WI and T1CE; Figure 4B), these families were more consistently recurrent across outer cross-validation folds, indicating a more concentrated and reproducible selection pattern. In ADC, the most stable families were land_d1_l2, land_d2_l0, and life_std_d2, each selected in all 5 runs (Figure 4D). Overall, these findings suggest that the apparent variability of ADC at the individual-feature level was largely attributable to substitutions among bin-level features within the same feature family, while its family-level representation remained highly reproducible.

### 3.3 Predictive performance of TDA, RAD, and TDA-RAD models across classifiers

**FIGURE 5:**
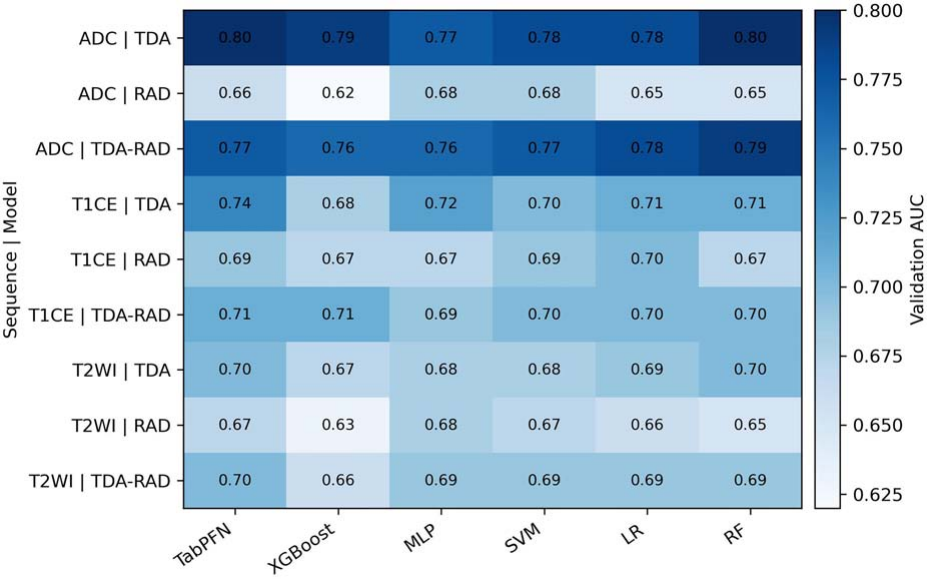
Predictive performance of TDA, RAD, and TDA-RAD models across classifiers and MRI sequences.

As shown in Figure 5, the validation AUCs of the TDA, RAD, and TDA-RAD models varied across MRI sequences and classifiers. Overall, models incorporating topological features generally showed better validation performance than RAD models across most sequence-classifier combinations. ADC-based models, particularly the ADC-based TDA model, demonstrated the strongest and most consistent discriminative ability among the three sequences, with the highest validation AUC reaching 0.80. In contrast, RAD models generally yielded weaker validation performance, while the TDA-RAD models failed to consistently outperform the corresponding TDA models, suggesting limited additional value from direct feature combination in the present study.

Across classifiers, TabPFN demonstrated the strongest overall performance, particularly in the TDA-based models. Therefore, TabPFN was selected as the primary classifier for subsequent analyses, and the sequence-specific performance of TDA models was further examined in detail.

### 3.4 Predictive performance of topological models across MRI sequences

As summarized in Table 2, the ADC-based TDA model showed the best validation overall performance. In the training set, the ADC-based model yielded a mean AUC of 0.87 (95% CI, 0.84–0.90). Importantly, it outperformed the other sequence-based models in the validation set, achieving the highest mean AUC of 0.80 (95% CI: 0.71–0.89), a mean accuracy of 0.73, and balanced mean sensitivity (0.70) and mean specificity (0.77). In contrast, although the T1CE-based model achieved the highest mean AUC in the training set (0.88, 95% CI: 0.87–0.89), its performance was less consistent in the validation set, where the mean AUC decreased to 0.74 (95% CI: 0.68–0.80). The T2WI-based model yielded the lowest overall performance, with a mean AUC of 0.84 (95% CI: 0.80–0.88) in the training set and 0.70 (95% CI: 0.57–0.83) in the validation set.

**TABLE 2:** Predictive performance of topological models across MRI sequences with TabPFN.

Sequence	Models	Dataset	AUC (95%CI)	Accuracy	Sensitivity	Specificity
ADC	TDA	Training	0.87 (0.84, 0.90)	0.81	0.78	0.84
	TDA	Validation	0.80 (0.71, 0.89)	0.73	0.70	0.77
T1CE	TDA	Training	0.88 (0.87, 0.89)	0.81	0.8	0.81
	TDA	Validation	0.74 (0.68, 0.80)	0.72	0.70	0.74
T2WI	TDA	Training	0.84 (0.80, 0.88)	0.75	0.71	0.79
	TDA	Validation	0.70 (0.57, 0.83)	0.68	0.66	0.70

Pairwise comparisons using DeLong’s test confirmed that the differences in AUC among all models were statistically significant. In the validation set, the ADC-based model demonstrated a significantly higher AUC than both the T1CE-based model (*P* < 0.001) and the T2WI-based model (*P* < 0.001). Additionally, the T1CE-based model showed a superior AUC compared to the T2WI-based model (*P* < 0.001).

### 3.5 Predictive performance of the clinical model and the combined topological-clinical model

Selected clinical characteristics with statistically significant between-group differences were used to construct the clinical model. As shown in Table 3, the clinical model showed moderate validation performance, with an AUC of 0.67 (95% CI, 0.49–0.84). After combining these characteristics with ADC-based topological features, the ADC-based topological-clinical model achieved a validation AUC of 0.80 (95% CI, 0.71–0.88). However, this performance did not exceed that of the ADC-based TDA model alone, suggesting limited incremental value from the selected clinical characteristics.

**TABLE 3:** Predictive performance of the clinical model and the ADC-based topological-clinical model.

Model	Dataset	AUC (95%CI)	Accuracy	Sensitivity	Specificity
Clinical	Training	0.74 (0.69, 0.78)	0.67	0.67	0.67
Clinical	Validation	0.67 (0.49, 0.84)	0.62	0.61	0.63
Topological-clinical	Training	0.90 (0.87, 0.94)	0.84	0.80	0.89
Topological-clinical	Validation	0.80 (0.71, 0.88)	0.75	0.73	0.76

### 3.6 Model performance assessment using ROC, calibration, and decision curve analysis

**FIGURE 6:**
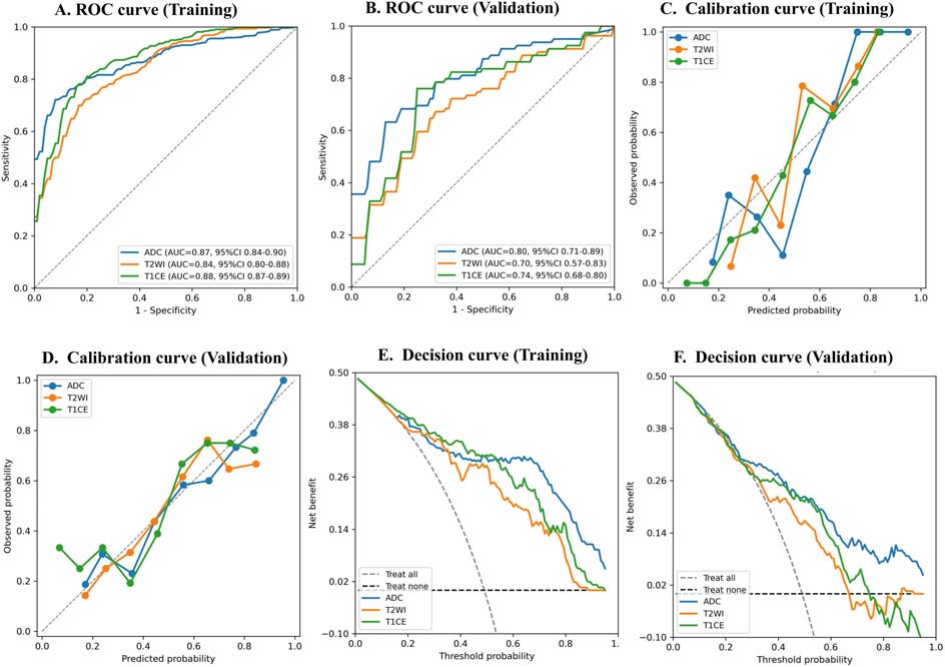
ROC, calibration, and decision curve analyses of topological models in training and validation sets. **(A,B)** ROC curves in the training and validation sets, respectively. **(C,D)** Calibration curves in the training and validation sets, respectively. **(E,F)** Decision curve analyses in the training and validation sets, respectively.

Given that models using topological features outperformed those using radiomics features across MRI sequences, and that TabPFN achieved the best overall predictive performance among the evaluated classifiers, subsequent performance evaluation focused on the TDA-based TabPFN models. The predictive performance of TabPFN models was assessed using ROC analysis, calibration curves, and decision curve analysis (Figure 6). In the validation set, ROC analysis showed good discrimination for the ADC-based model, while the models derived from T1CE and T2WI showed lower discrimination. Calibration curves indicated that all three sequence-specific models were well calibrated during training (Figure 6C) and remained well calibrated overall in validation, with the ADC-based model aligning most closely with the 45-degree reference line (Figure 6D). Decision curve analysis showed that, in the validation cohort, the ADC-based model provided a higher net benefit than both the full-intervention and none-intervention strategies across a broad threshold range of approximately 0.27–0.98 (Figure 6F). The corresponding main clinically beneficial threshold ranges were narrower for the T1CE-based model (approximately 0.30–0.74) and the T2WI-based model (approximately 0.28–0.66).

### 3.7 Interpretability analysis

**FIGURE 7:**
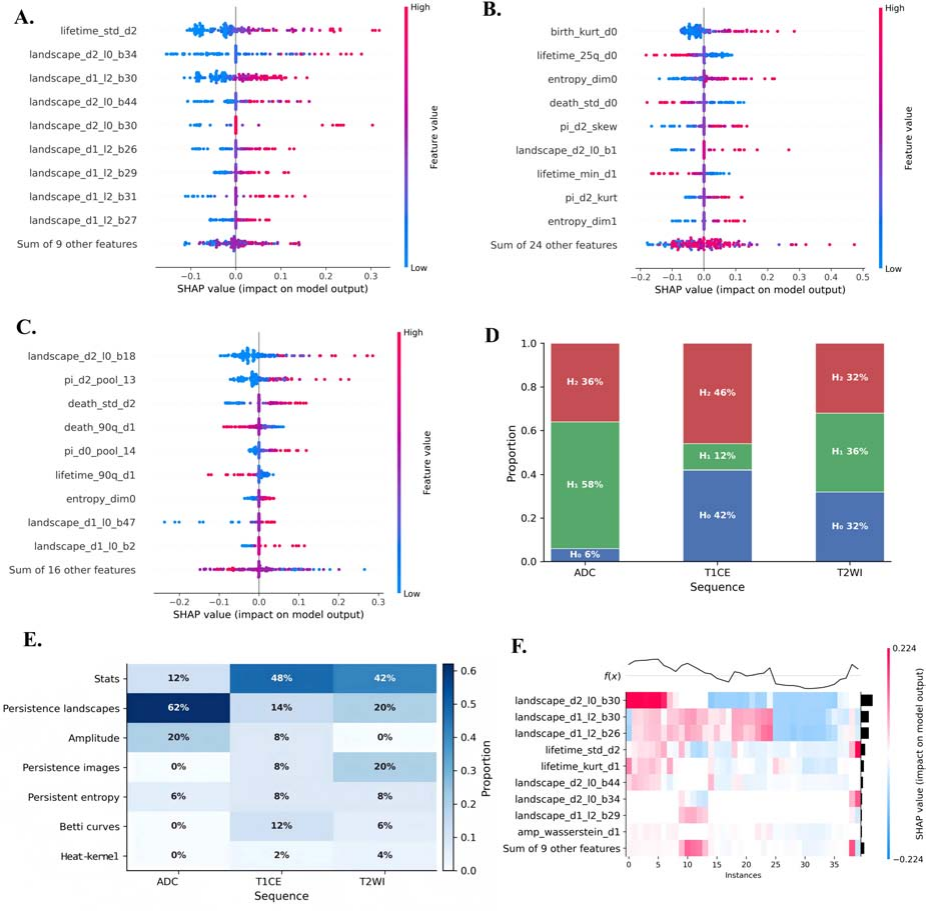
SHAP interpretability analysis of topological features across MRI sequences. **(A–C)** SHAP beeswarm plots of the top 10 features for the ADC-based, T1CE-based, and T2WI-based models. **(D)** Distribution of selected features across homology dimensions (H_0_, H_1_, and H_2_). **(E)** Frequency of selected descriptor families across sequences. **(F)** SHAP heatmap showing patient-level contribution patterns in the ADC-based model.

Given its best overall performance, the ADC-based TDA model under TabPFN was selected for SHAP analysis to further interpret the contributions of topological features. Figures 7A–C summarize the top 10 features for each MRI sequence ranked by mean absolute SHAP values, illustrating their contributions to the prediction of EHETR and non-EHETR. Higher absolute SHAP values reflect greater feature influence. More specifically, in the ADC sequence (Figure 7A), the top three predictive features were lifetime_std_d2 (H_2_ dimension), landscape_d2_l0_b34 (H_2_ dimension), and landscape_d1_l2_b30 (H_1_ dimension). In the beeswarm plot, higher values of these features were generally associated with positive SHAP values, indicating that increases in these features tended to increase the predicted probability of EHETR; this pattern was particularly evident for the H_1_ persistence landscape feature landscape_d1_l2_b30. A positive SHAP value for a specific H_2_ persistence landscape feature indicates that an increase in this feature value significantly elevates the probability of EHETR occurrence. In the T2WI and T1CE models (Figures 7B,C), the SHAP contribution patterns were more heterogeneous, with both positive and negative effects observed among the leading features.

To assess the consistency of these findings across cross-validation folds, we aggregated the top 10 features within each fold by homology dimension (H_0_, H_1_, and H_2_) and feature type to compare their relative contributions. As shown in Figure 7D, selected features spanned all three homology dimensions, with H_1_ and H_2_ persistence landscape features contributing most frequently across folds (ADC: H_0_, 6% H_1_, 58%, H_2_, 36%; T1CE: H_0_, 46% H_1_, 12%, H_2_, 42%; T2WI: H_0_, 32% H_1_, 36%, H_2_, 32%), highlighting the importance of higher-order topological features. For the distribution of feature types shown in Figure 7E, persistence landscape features accounted for the largest proportion of all selected features, comprising approximately 32%. When stratified by MRI sequence, persistence landscape features were most prevalent in the ADC sequence (62%).

Patient-level contribution patterns were further visualized using SHAP heatmaps (Figure 7F) based on the ADC-based model, which achieved the highest AUC among sequences. The heatmap showed structured bands of positive and negative SHAP values for persistence landscape features across patients, along with differences in predicted probabilities between EHETR and non-EHETR cases.

## 4 Discussion

In this study, 161 patients with meningiomas were included, and 79 (49.1%) developed EHETR after surgical resection. Our findings demonstrated that the ADC-based model using topological features extracted from the PE region achieved the highest mean validation AUC of 0.80, significantly outperforming the corresponding models based on T1CE and T2WI. SHAP analysis revealed that persistence landscape features in the H_1_ (loops) and H_2_ (cavities) homology dimensions were the primary drivers of predictive models for EHETR in meningiomas. To our knowledge, this is the first study to apply TDA to the prediction of postoperative complications in brain tumors.

The results of this study demonstrate that topological features from the PE region are feasible for predicting EHETR in meningiomas, especially when derived from ADC maps. ADC provides a quantitative measurement of water-proton movement in cellular spaces (27) and is more sensitive to changes in the water content of cerebral parenchyma than T1CE and T2WI imaging. Accordingly, within the PE region, ADC is more sensitive to brain tissue damage subsequent to the vasogenic edema (28–30). The pathological characteristics are complex, beyond vasogenic edema, they are associated with blood-brain barrier dysfunction, extracellular fluid accumulation, and heterogeneous tumor-brain interactions (31, 32). Accordingly, ADC-based topological features may better reflect edema severity and spatial extent; this facilitates models built on these signatures to achieve superior EHETR prediction. Hu et al. (4) reported results similar to ours, demonstrating that ADC features of PE are associated with postoperative progressive cerebral edema and hemorrhage. Together with previous evidence linking PE to postoperative complications, these findings further support the important role of PE in the development of EHETR (3, 4, 33). More importantly, ADC-derived topological features from the PE region may provide a novel tool and a new direction for exploring the mechanisms underlying EHETR.

**FIGURE 8:**
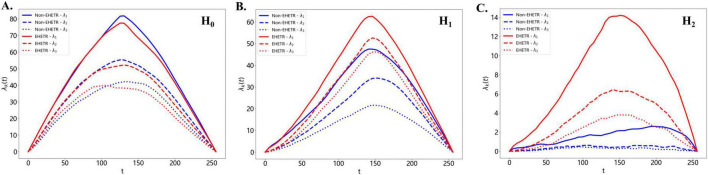
Persistence landscape curves from ADC based model in EHETR and non-EHETR groups. **(A)** Persistence landscapes in the H_0_ dimension. **(B)** Persistence landscapes in the H_1_ dimension. **(C)** Persistence landscapes in the H_2_ dimension.

Persistent homology characterizes topological structures across different homology dimensions, with H_0_ representing connected components, H_1_ representing loop-like structures, and H_2_ representing cavity-for like structures. Based on the SHAP analysis, the ADC sequence showed a clearly higher-order topological pattern, with 94% of the top-ranked features originating from the H_1_ and H_2_ homology dimensions, suggesting that EHETR-related information was mainly captured by higher-order topological variation. At the descriptor-family level, persistence landscape features accounted for 62% of the important features in the ADC model. Persistence landscape converts the birth, death, and persistence of topological structures into a quantitative functional representation that captures their strength, stability, and distribution across scales. Consistent with these findings, the group-wise comparisons shown in Figure 8 suggested that the between-group differences were more evident in the H_1_ and H_2_ persistence landscapes, whereas the distinction in H_0_ appeared relatively weaker. The generally higher and broader landscape curves in the H_1_ and H_2_ dimensions, together with the consistently elevated profiles of the EHETR group across multiple layers (λ_1_, λ_2_, and λ_3_), suggest more prominent higher-order topological variation and multiscale structural complexity within the PE region. This pattern further suggests that the between-group difference may reflect distributed multiscale topological variation rather than a change confined to a single dominant feature. These findings support that PE plays an important role in the development of EHETR, which is consistent with previous studies (7, 8), and suggest that ADC-derived topological features associated with the severity and extent of PE can provide additional information and clues for exploring the mechanism of EHETR.

In the present study, multiple classifiers were evaluated under the same experimental framework, and TabPFN showed the most favorable overall performance. This finding is in line with previous studies. The original study introducing TabPFN as a tabular foundation model showed that it achieves superior predictive accuracy compared with conventional machine learning approaches, particularly in low-data settings (24). Two subsequent studies (34, 35) applied TabPFN to medical imaging cohorts of 218 and 276 patients, respectively, and compared it with conventional machine learning classifiers. In these studies, TabPFN was compared with multiple classifiers and was reported to perform best in several model comparisons. Overall, these results suggest that TabPFN is particularly suitable for medical imaging studies with limited sample sizes and should be further investigated and validated in future clinical applications.

The decision curve analysis demonstrated that the ADC-based model provided a higher net benefit across a wide threshold probability range (0.27–0.98), indicating its potential utility in diverse clinical decision-making scenarios. At lower threshold probabilities, the risk of EHETR is relatively low, suggesting that routine postoperative management can be maintained and unnecessary interventions can be avoided. At higher threshold probabilities, the model may support more aggressive interventions. In addition, the AUC value of our TDA model was 0.8. As the AUC value represents the overall discriminative ability of a clinical model, where a value of 0.5 indicates no diagnostic utility, 0.7–0.8 is considered acceptable, and 0.8–0.9 is classified as excellent performance (36). Therefore, the performance of AUC value of 0.8 in the present study is relatively sufficient to support the application of ADC-based clinical thresholds in the management of EHETR. It may aid in risk stratification by identifying patients at high risk of EHETR, which can subsequently improve more precise and personalized treatment, including surgical planning and ICU monitoring decisions, ultimately enabling the optimization of patient care.

This study has several limitations. Our study was limited by a relatively small sample size and the inclusion of patients from a single center only, limiting the generalizability of the proposed model across different institutions, scanners, and imaging protocols. In addition, the biological interpretability of the broad range of topological features employed in this study remains limited, which may hinder their direct clinical interpretation. Although TabPFN performed well with small samples, its application in medical imaging analysis is still limited and warrants further investigation.

## 5 Conclusion

In the present study, the topological features demonstrated multi-scale structural organization of meningiomas, and the persistent homology based topological features derived from the PE region hold promise for preoperative predicting of EHETR in meningiomas as a novel approach. SHAP analysis highlighted key contributions from higher order topological descriptors, particularly H_1_ and H_2_ persistence landscapes. These findings suggest that topological characterization of PE may provide new insights for mechanistic exploration of EHETR in meningiomas. Future multicenter prospective studies are warranted to further validate the proposed model and facilitate its potential clinical translation, while further pathophysiological investigations may help clarify the biological significance of the core topological features.

## Data Availability

The original contributions presented in the study are included in the article. Further inquiries can be directed to the corresponding authors.
